# Biting mechanics and niche separation in a specialized clade of primate seed predators

**DOI:** 10.1371/journal.pone.0190689

**Published:** 2018-01-11

**Authors:** Justin A. Ledogar, Theodora H. Y. Luk, Jonathan M. G. Perry, Dimitri Neaux, Stephen Wroe

**Affiliations:** 1 Zoology Division, School of Environmental and Rural Science, University of New England, Armidale, New South Wales, Australia; 2 Center for Functional Anatomy and Evolution, Johns Hopkins University, Baltimore, Maryland, United States of America; Monash University, AUSTRALIA

## Abstract

We analyzed feeding biomechanics in pitheciine monkeys (*Pithecia*, *Chiropotes*, *Cacajao*), a clade that specializes on hard-husked unripe fruit (sclerocarpy) and resistant seeds (seed predation). We tested the hypothesis that pitheciine crania are well-suited to *generate* and *withstand* forceful canine and molar biting, with the prediction that they generate bite forces more efficiently and better resist masticatory strains than the closely-related *Callicebus*, which does not specialize on unripe fruits and/or seeds. We also tested the hypothesis that *Callicebus*-*Pithecia*-*Chiropotes*-*Cacajao* represent a morphocline of increasing sclerocarpic specialization with respect to biting leverage and craniofacial strength, consistent with anterior dental morphology. We found that pitheciines have higher biting leverage than *Callicebus* and are generally more resistant to masticatory strain. However, *Cacajao* was found to experience high strain magnitudes in some facial regions. We therefore found limited support for the morphocline hypothesis, at least with respect to the mechanical performance metrics examined here. Biting leverage in *Cacajao* was nearly identical (or slightly less than) in *Chiropotes* and strain magnitudes during canine biting were more likely to follow a *Cacajao*-*Chiropotes*-*Pithecia* trend of increasing strength, in contrast to the proposed morphocline. These results could indicate that bite force efficiency and derived anterior teeth were selected for in pitheciines at the expense of increased strain magnitudes. However, our results for *Cacajao* potentially reflect reduced feeding competition offered by allopatry with other pitheciines, which allows *Cacajao* species to choose from a wider variety of fruits at various stages of ripeness, leading to reduction in the selection for robust facial features. We also found that feeding biomechanics in sympatric *Pithecia* and *Chiropotes* are consistent with data on food structural properties and observations of dietary niche separation, with the former being well-suited for the regular molar crushing of hard seeds and the latter better adapted for breaching hard fruits.

## Introduction

Diet may be an important selective pressure that has influenced the evolution of many primate clades. Fossil primates, including but not limited to australopithecines, sub-fossil lemurs, adapiforms, early anthropoids, and fossil cercopithecoid monkeys, have all been hypothesized to possess craniofacial adaptations for feeding on resistant foods [1–5]. Such adaptations are predicted to include those that increase bite force and strengthen the face [6, 7]. However, a more complete understanding of dietary adaptations in living organisms is necessary in order to understand the evolution of such adaptations in extinct taxa [8]. Here we examine the relationship between diet and feeding biomechanics in the Pitheciinae, a clade of extant primates that consume resistant foods but that exhibit variation in both morphology and feeding ecology [9, 10].

Pitheciines are a monophyletic subfamily of Neotropical seed predators that includes the sakis (*Pithecia*), bearded sakis (*Chiropotes*), and uakaris (*Cacajao*). Pitheciine monophyly has been well-established by both molecular [11, 12] and morphological [13, 14] studies, with *Pithecia* being the sister taxon to a clade including *Chiropotes* and *Cacajao*. Some molecular phylogenies further support the inclusion of *Callicebus* (titi monkeys) as the most basal pitheciine [15], but most recent studies group them with the pitheciines at the family level as the Pitheciidae [16–19]. Pitheciines, and in particular *Chiropotes* and *Cacajao*, exhibit a suite of highly derived cranial, mandibular, and dental morphologies hypothesized to be adaptations for feeding on unripe fruits and seeds [10, 20–22]. Their specialization on resistant foods, close-relatedness, and observed interspecific variation in feeding ecology (see below) make pitheciines an ideal model clade for testing hypotheses related to feeding biomechanics and dietary adaptation in primates.

Extant pitheciines are among the most frugivorous of all New World monkeys, with fruit composing up to 90% of the diet [9, 23–26]. Unlike most primate species, all pitheciines rely heavily on unripe fruits with thick, resistant husks, as opposed to those that have matured and softened [9, 24–29]. Unripe fruits are breached by pitheciines in order to gain access to nutrient-rich seeds [10, 30], a type of frugivory referred to as “sclerocarpic harvesting” [9]. Preying upon the seeds of unripe fruits appears to represent a novel means of acquiring necessary nutrients, including lipids and fiber [31]. As fruit ripens and becomes softer, the seeds harden, and develop higher levels of toxic secondary compounds [9, 24, 27]. All pitheciines eat seeds throughout the year, as opposed to falling back on them seasonally, and there is no particular relationship between fruit pericarp hardness and seasonal rainfall [29, 32, 33].

Relative to closely-related taxa (e.g., *Callicebus*), pitheciines exhibit a suite of derived craniodental features related to their unique dietary strategy. In particular, the anterior teeth are well-suited to exploit hard-husked fruits [10]. Sclerocarpic harvesting in pitheciines first involves a forceful gouge through the pericarp of an unripe fruit using large, robust, wedge-shaped canines. The canines of *Chiropotes* and *Cacajao* are exceptionally large and robust [10, 21], with buccolingual tapering that creates well-developed cutting edges [34]. This wedge-like morphology reduces wear and conserves muscle force by facilitating crack propagation in the opposing food [35]. In addition, pitheciine canines splay laterally from the incisal and postcanine tooth rows, which reduces interference from the incisors during fruit puncture [10]. Fruit mesocarp and seeds are then scooped from the inside of fruit husks using narrow and procumbent incisors [10, 14].

Specialized canines allow pitheciines to extract nutrient-rich seeds from inside unripe fruits before being processed by the postcanine teeth. The seeds masticated by pitheciines are elastic and highly fibrous [30], but also exhibit high crushing resistance [9, 25, 26, 32]. They can therefore be described as being “hard and resilient” [10] as opposed to the hard and brittle seeds crushed by other primate seed predators (e.g., *Cercocebus*; [36]). To overcome such mechanical defenses, pitheciine molars and premolars are well-suited for crushing and grinding behaviors. Compared with those of *Callicebus* and other New World primates, pitheciines exhibit virtually no postcanine cresting and have low molar cusps, yet the occlusal surfaces are highly crenulated [10, 37]. These crenulations increase the number of occlusal surface features exhibited by pitheciine molars [22], which facilitate secondary breakdown of seed particles [38] and help position seeds during mastication [35]. Although pitheciines have relatively thin enamel, they exhibit strong molar enamel prism decussation to defend the enamel against crack propagation [39].

Kinzey [10] suggested that *Callicebus*-*Pithecia*-*Chiropotes*-*Cacajao* represent a morphocline of increasing anterior dental specialization for the husking of unripe fruits with hard pericarps for the purposes of preying on their seeds. Although *Callicebus* is not typically regarded as a “seed predator,” some species (e.g., *C*. *torquatus*) are known to include a high proportion of seeds in their diet [40, 41]. Correspondingly, titis lack the highly derived anterior dental condition exhibited by *Pithecia*, *Chiropotes*, and *Cacajao*, but they have relatively tall incisors and reduced molar relief relative to ripe-fruit feeding taxa that do not consume seeds, such as *Aotus* [10, 22, 37]. Dietary data are limited for *Cacajao*, but material property data collected on foods eaten by *Pithecia* and *Chiropotes* reveal important differences in feeding ecology that are consistent with Kinzey’s [10] proposed morphocline.

*Pithecia* and *Chiropotes* are broadly sympatric and are most commonly found in non-flooded forests, whereas *Cacajao* is mainly found in seasonally flooded forests [27, 42–44]. Given the notion of niche partitioning, diets are expected to differ most between sympatric congeneric primate species [45]. Several authors have discussed the importance of differential habitat preference and dietary niche separation in sympatric *Chiropotes* and *Pithecia* [9, 26, 46]. For example, *Chiropotes satanas* and *Pithecia pithecia* are able to remain sympatric and reduce competition by exploiting fruits at different stages of ripeness. Mechanical property studies of fruits and seeds eaten by pitheciines [9, 26, 32] demonstrate that the younger fruits breached by *Chiropotes* have higher puncture resistance than fruits exploited by *Pithecia*. This is reflected by a relatively more robust canine in *Chiropotes* [10, 21], as well as increased canine root surface area [47]. *Chiropotes* has also been shown to have greater mechanical efficiency (i.e., leverage) for the jaw adductors compared to *Pithecia* at both molar and anterior tooth bite points [21, 31, 48]. Bite force efficiency is thought to be adaptively significant in primates [1, 6, 7, 49] and other mammalian groups (e.g., bats [50]), with species that consume foods requiring forceful biting (e.g., hard fruits or seeds) expected to exhibit greater leverage. An increased ability to efficiently generate masticatory force in pitheciines is matched by an apparent increased ability to resist stress and strain, as several studies [20, 21, 31] have found that the mandible of *Chiropotes* is relatively more robust than that of *Pithecia*.

The ingestion of unripe fruit pericarp appears to be a selectively important behavior for the pitheciine clade. It has been suggested that species relying heavily on ingestive behaviors (e.g., *Chiropotes*, *Cercocebus*) should exhibit craniofacial adaptations that reduce strains in the rostrum [1, 2]. For example, the ingestion of mechanically challenging foods may have been selectively important for early hominins [1, 51]. A finite element analysis (FEA) of feeding biomechanics in *Australopithecus africanus* by Strait *et al*. [2] found that the characteristic “anterior pillars” of this fossil hominin species act to resist compressive strains during forceful premolar loading, such as when cracking open a hard seed or nut. These strains become highly elevated in simulations where the pillar is removed or reduced in size [52]. The use of FEA to the study of functional morphology in fossil hominins [2, 7, 53, 54] is still relatively new and may yield important insight into the ecology of extinct species. However, a goal of future research should be to gain a broader understanding of the application of modeling techniques to the study of craniofacial biomechanics and diet in living primates (e.g., [55–58]).

We examine feeding biomechanics in the Pitheciinae (*Pithecia*, *Chiropotes*, *Cacajao*) relative to the only other member of the Pitheciidae, *Callicebus*. Simulations of forceful canine biting (as when breaching an unripe fruit) and molar biting (as when crushing a resistant seed) were performed using FEA. Previous analyses of biting mechanics in pitheciines [21, 31, 48] have relied on photographs and 2D estimates of lever/load arms, using methods that consider only the anterior-most cranial attachment of each muscle [59]. Our 3D analysis of feeding biomechanics considers entire muscle origins, as well as the orientation of muscle vectors. FEA also facilitates comparisons of structural strength. Specifically, we test the hypothesis (Hypothesis 1) that pitheciines are well-suited to *generate* and *withstand* forceful canine and molar biting, with the prediction that pitheciine species will exhibit greater bite force leverage and capacity to resist masticatory strain relative to *Callicebus*. We further test the hypothesis (Hypothesis 2) that *Callicebus*-*Pithecia*-*Chiropotes*-*Cacajao* represent a morphocline of increasing sclerocarpic specialization [10], with the prediction that these species will follow a trend of increasing bite force leverage and craniofacial strength.

## Materials and methods

### Selection of specimens for FEA

Smith *et al*. [57] show that intraspecific differences in craniofacial shape can result in high levels of variation in strain magnitudes during feeding, but that the distribution of strain concentrations is relatively conserved within species. To partially account for cranial shape variation within each of *Callicebus*, *Pithecia*, *Chiropotes*, and *Cacajao*, we collected 3D landmark data using Checkpoint software (Stratovan) from a sample of 21 pitheciid (i.e., Callicebinae + Pitheciinae) specimens (Table 1) surface-rendered using CT image stacks available on the MorphoSource (www.morphosource.org), DigiMorph (www.digimorph.org), and Primate Research Institute (dmm.pri.kyoto-u.ac.j) databases. Only fully adult specimens preserving the mandible and free of damage (i.e., suitable for FEA) were included in this shape analysis. A formalin-fixed cadaveric head of a male *Chiropotes satanas* (courtesy of J. Fleagle, Stony Brook University) was also included in the sample. This specimen was also used to collect jaw adductor muscle forces (see below).

**Table 1:** Pitheciid specimens included in the analysis of cranial shape. MCZ = Museum of Comparative Zoology (Harvard); USNM = United States National Museum (Smithsonian); M = male; F = female; U = sex unknown.

Taxon	Specimen No.	Sex
*Callicebus moloch*	MCZ 26922	M
*Callicebus moloch*^1^	MCZ 30564	M
*Callicebus moloch*	MCZ 30566	M
*Callicebus moloch*^1^	MCZ 32383	M
*Callicebus moloch*	MCZ 37828	M
*Pithecia monachus*	MCZ 20266	U
*Pithecia monachus*	MCZ 27124	U
*Pithecia pithecia*^1^	MCZ 30719	F
*Pithecia monachus*^1^	MCZ 30720	F
*Pithecia pithecia*	MCZ 31061	M
*Chiropotes albinasus*	MCZ 31701	F
*Chiropotes satanas*	Stony Brook	M
*Chiropotes satanas*	USNM 338964	M
*Chiropotes satanas*^1^	USNM 388168	M
*Chiropotes satanas*	USNM 406588	M
*Chiropotes satanas*^1^	USNM 518225	M
*Chiropotes satanas*	USNM 549519	F
*Cacajao calvus*	MCZ 1957	U
*Cacajao calvus*^1^	USNM 302626	M
*Cacajao calvus*^1^	USNM 302627	F
*Cacajao calvus*	USNM 319516	M

^1^Specimen selected for FEA.

**Fig 1:**
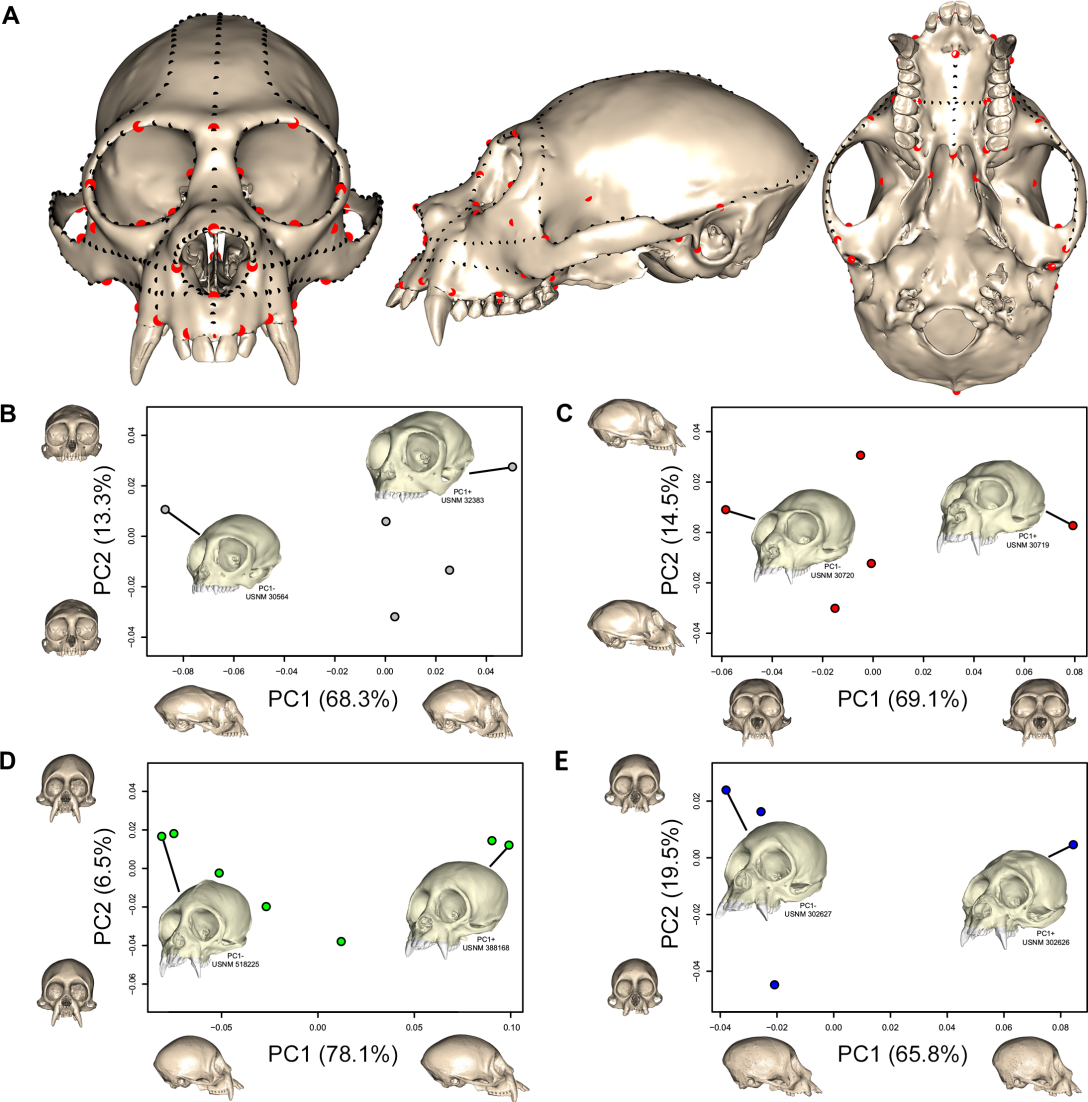
Shape analysis. (**A**) Cranium of *Pithecia pithecia* (MCZ 30719) in anterior, lateral, and inferior view showing fixed landmarks (red) and sliding semi-landmarks (black) used in the shape analysis. Plots show results for the PCA of cranial shape in (**B**) *Callicebus*, (**C**) *Pithecia*, (**D**) *Chiropotes*, and (**E**) *Cacajao*. For each plot, crania at the positive and negative ends of the PC1 and PC2 axes describe shape change along these axes, warped onto the specimen closest to the group centroid (i.e., shortest distance from group centroid). The specimens with the most positive and most negative principal component scores along PC1 were selected for use in FEA. In *Callicebus*, PC1 mainly captures variation in neurocranial height and length and facial length, with longer and lower crania with slightly longer faces toward negative scores. PC2 explains some differences in facial width and orbit, with wider faces toward minimum scores. In *Pithecia*, PC1 describes differences in facial and neurocranial width, with wider crania toward positive scores. PC2 reflects aspects of neurocranial and facial variation, with the neurocranium extended superoinferiorly and face extended inferoanteriorly toward positive scores. In *Chiropotes*, PC1 captures some differences in neurocranial shape and facial projection, with longer crania and faces toward positive scores. Faces are slightly wider toward positive scores along PC2. *Cacajao* is similar to *Chiropotes*, except that longer crania and face are found along the negative end of PC1.

The landmark configuration included 51 fixed landmarks and 365 sliding semilandmarks along 22 curves (Table 2). Semilandmarks were permitted to slide along homologous curves based on minimized bending energy and were thus also considered homologous [62]. Shape coordinates of the landmarks were subjected to Generalized Procrustes Analysis (GPA) to ensure that shape was the only variable among specimens by translating the centers of the models to the same origin, scaling them to a common size, and rotating them to a best-fit using a least-squares calculation [63, 64]. The Procrustes coordinates were then analyzed using Principal Component Analysis (PCA) using the R [65] “Geomorph” package [66]. The purpose of this analysis was to account for variation within each of the four groups examined, so we performed separate PCAs for each group and chose the two specimens with the most positive (PC1+) and most negative (PC1-) principal component scores along the first axis (Fig 1). These specimens were then used to build eight finite element models (FEMs). It should be noted that our analysis of intraspecific shape variation used in choosing specimens for FEA is limited by small sample sizes and that we have not comprehensively assessed shape variation in these species. It is therefore possible that our biting simulations do not capture the full range of mechanical variation within pitheciids. However, high levels of intraspecific shape variation are not always associated with high levels of mechanical variation [67] and it is not uncommon for studies such as the present one to include single specimens per species (e.g., [53, 55, 68]), due to the time-consuming nature of model construction. Nonetheless, we have attempted to account for intraspecific shape variation by including the two most distinct crania per group from among the previously-scanned specimens available to us.

**Table 2:** Landmarks and semilandmarks included in the shape analysis. Landmark definitions follow White *et al*. [60] and Baab [61]).

Fixed Landmarks		Curves	Curve definition	SLM^1^
Alare (L, R)	Lateral squamotympanic fissure (L, R)	Superior sagittal	Rhinion to inion	30
Ant. articular surface (L, R)	Mid-torus inferior (L, R)	Nasoalveolar clivus	Nasospinale to prosthion	5
Auriculare (L, R)	Nasospinale	Nasal margin (L, R)	Rhinion to nasospinale	15
C1/P2 (L, R)	Inion	Lateral orbital margin (L, R)	Mid-torus inf. to orbitale—lateral	10
Dacryon (L, R)	Incisivion	Medial orbital margin (L, R)	Mid-torus inf. to orbitale—medial	10
Ectoconchion (L, R)	Orbitale (L, R)	Canine root (L, R)	I2/C1 to lacrimale	10
Ectomolare—M3 (L, R)	Postglenoid process (L, R)	Temporal fossa (L, R)	Jugale to auriculare^2^	50
Ectomolare—P4/M1 (L, R)	Prosthion	Inferior zygomatic arch (L, R)	Lat. squamotympanic fissure to zygomatic root	15
Endomolare—M3 (L, R)	Pterygoid hamulus (L, R)	Superior zygomatic arch (L, R)	Jugale to auriculare^3^	15
Endomolare—P4/M1 (L, R)	Rhinion	Midface (L, R)	Jugale to alare	15
Glabella	Staphylion	Lower face (L, R)	Zygomatic root to nasospinale	15
I1/I2 (L, R)	Temporosphenoid suture (L, R)	Sagittal palate	Incisivion to staphylion	10
I2/C1 (L, R)	Zygomatic root (L, R)	Transverse palate	Endomolare P4/M1 to endomolare P4/M1	10
Jugale (L, R)	Zygomaticofacial foramen (L, R)			
Lacrimale (L, R)				

^1^Number of semilandmarks defined along a curve.

^2^Following the temporal lines and the lateral orbit.

^3^Along the zygomatic arch.

### Finite element model construction

CT data on the eight specimens chosen for analysis were used to generate solid (volumetric) meshes using a combination of thresholding in Mimics v 18.0 (Materialise, Ann Arbor, MI, USA), surface editing in Geomagic Studio 2014 (Research Triangle Park, NC, USA), and solid-meshing in 3-Matic v 10.0 (Materialise, Ann Arbor, MI, USA), largely following the methods outlined by Smith *et al*. [7, 57]. Differences in scan resolution and therefore the quality of the models were reduced before solid-meshing by first re-meshing the surfaces using a target polygon edge length of 0.5 mm. This resulted in solid models of similar mesh density (between 1,236,492 in the smaller *Callicebus* and up to 2,055,744 elements in *Cacajao*). We created separate volumes of 4-noded tetrahedral (tet4) elements for each of the teeth, with each tooth consisting of one volume for the pulp cavity nested within a volume for the dental tissue (dentine + enamel). A periodontal ligament (PDL) was also included for each tooth by creating thin volumes (0.2 mm [69]) between the alveoli and tooth roots. Trabecular density in the species included in our sample was low, so we chose not to model whole volumes of trabeculae nested within the cortical bone volume (e.g., [7, 54, 57, 67]). However, we did model trabecular vacuities in the maxillary and zygomatic regions, as opposed to modeling these areas as solid bone.

Solid models were imported as Nastran (NAS) files into Strand7 (Strand7 Pty Ltd, NSW, Sydney, Australia) FEA software. The separate volumes for bone, teeth, and PDL were “zipped” to form a contiguous mesh. An enamel “cap” was also created during this stage by selecting surface elements on the tooth crowns and assigning them to their own group and set of properties. As discussed below, our comparisons of craniofacial stiffness (strength) focus on shape-related mechanical differences, so we assigned all models the same set of homogeneous and isotropic properties from the literature (Table 3). Muscle forces were also scaled as to remove the effects of size from the mechanical results (see below).

**Table 3:** Material properties. Elastic (Young’s) moduli (*E*) and Poisson’s ratios (*v*) assigned to finite element models.

Material	*E* (MPa)	*v*	Reference
Cortical bone	17,319	0.28	Strait *et al*. [2]
Enamel	84,100	0.3	Magne [70]
Dentin	18,600	0.31	Ko *et al*. [71]
Pulp	2	0.45	Rubin *et al*. [72]
PDL	68.9	0.45	Holmes *et al*. [73]

### Muscle forces and constraints

Jaw adductor muscle forces were applied to each FEM for the temporalis, masseter, and medial pterygoid under the assumption that the chewing muscles were acting at peak activity levels on both sides of the cranium. These loads allow an estimate of the maximum bite force potential. As noted above, our comparisons focus on differences in mechanical performance that are purely the result of differences in shape. We removed the effects of size from the strain results by scaling forces based on differences in model volume [74]. The model of a male *Chiropotes* (PC1-) was chosen as the “baseline” for muscle force scaling. Baseline forces applied to this model were collected from a formalin-fixed cadaveric head of a male *Chiropotes satanas*, which was also included in the shape analysis described above.

**Fig 2:**
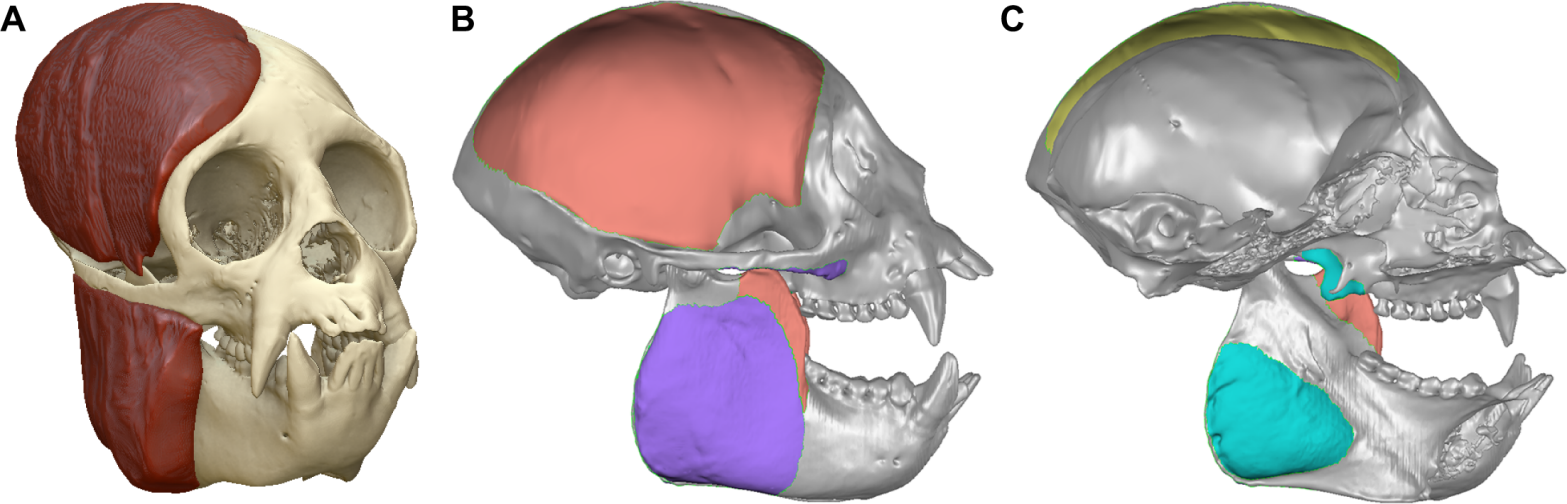
Digital dissection and jaw adductor muscle areas. (**A**) Surface model of *Chiropotes satanas* specimen showing digitally dissected temporalis and masseter muscles used to calculated forces applied to FEMs. (**B**) Lateral view of the *Chiropotes* PC1- surface model showing origin and insertion areas for the temporalis (red) and masseter (purple) muscles. (**C**) Sagittal section showing origin and insertion for the medial pterygoid (blue-green). Note that muscle tractions were only applied to origins (i.e., those attachments on the cranium), whereas the insertions (i.e., on the mandible) were used to guide the orientation of muscle force vectors.

We microCT scanned the cadaveric specimen of *Chiropotes* at Penn State’s Center for Quantitative Imaging and collected cross-sectional area (CSA) data on the temporalis and masseter muscles. The muscles on the left side of the head had been previously dissected, preserving an edge outlining the origin and insertion areas that helped with applying muscles to the FEMs. Much of the cervical region had also been dissected, with apparent damage to the pterygoid muscles. Before scanning, the skin and soft tissues overlying the temporalis and masseter muscles on the right side were freed, exposing the muscles. The masseter and temporalis muscles, as well as the skull, were then segmented from the CT images and converted into a 3D surface model (Fig 2A) using Mimics software. CSAs were then calculated in Geomagic Studio at the thickest part of each muscle, perpendicular to the primary orientation of the muscle fibers. Our CSAs were very similar to physiological cross-sectional areas (PCSAs) reported by Anapol *et al*. [75] for both temporalis (4.21 cm^2^ vs. 4.26 cm^2^) and masseter (2.00 cm^2^ vs. 2.08 cm^2^). Therefore, we used the proportional relationship between the primary three jaw adductors of *Chiropotes* reported by Anapol *et al*. [75] to calculate a CSA for the medial pterygoid in our specimen (1.54 cm^2^). CSAs were then used to calculate forces in Newtons (N) such that each cm^2^ of muscle was equivalent to 30 N [76]. The “baseline” model of *Chiropotes* PC1- was then assigned these unscaled forces, and the remaining models were applied total forces that were either scaled up or down based on differences in model volume to the two-thirds power (Table 4). This muscle force scaling procedure removes the effects of differences in model size on stress and strain from the mechanical results [74, 77].

**Table 4:** Muscle force scaling. Muscle forces in Newtons (N) were scaled by model size, where size is represented by model volume in mm^3^ to the two-thirds power.

Model	Volume	Batch^1^	Temporalis	Masseter	Med. pterygoid
*Callicebus* PC1+	5293.20	1	43.85	21.41	15.85
		2	40.55	26.36	14.19
*Callicebus* PC1-	5478.36	1	44.86	21.90	16.22
		2	41.49	26.97	14.52
*Pithecia* PC1+	10358.06	1	68.60	33.49	24.80
		2	54.43	44.41	28.05
*Pithecia* PC1-	8747.40	1	61.29	29.92	22.16
		2	48.63	39.68	25.06
*Chiropotes* PC1+	21711.81	1	112.35	54.86	40.62
*Chiropotes* PC1-^2^	26340.79	1	127.80	62.40	46.20
*Cacajao* PC1+	28574.96	1	134.93	65.88	48.78
		2	150.07	52.28	49.15
*Cacajao* PC1-	28905.45	1	135.97	66.39	49.15
		2	148.93	51.88	48.78

^1^FEMs of *Callicebus*, *Pithecia*, and *Cacajao* were run twice. The first batch (Batch 1) assigned models total muscle forces that were scaled from *Chiropotes* PC1-. For the second batch (Batch 2), total muscle forces were scaled following the same procedure, but forces were redistributed according to species-specific muscle force ratios [75, 78]. See main text for further details.

^2^Baseline FEM for muscle force scaling.

Although the FEMs were assigned comparable muscles forces by our scaling procedure, differences in jaw adductor muscle force ratios between species are likely to have an impact on mechanical performance. Notably, Anapol *et al*. [75] report a much greater temporalis/masseter PCSA ratio in *Chiropotes* (2.11) as compared to both *Pithecia* (1.23) and *Callicebus* (1.54). We measured a similarly high ratio in our cadaveric specimen (2.05). Anapol *et al*. [75] do not include *Cacajao*, but Cachel [78] reports an even larger temporalis/masseter ratio (2.87) based on dry weight measurements. Therefore, we performed a second batch (Batch 2) of biting simulations in order to account for differences in muscle force ratios by redistributing the total muscle forces in *Callicebus*, *Pithecia*, and *Cacajao* according to these data (see Table 4). Taylor *et al*. [79] also analyzed jaw muscle architecture in a sample of platyrrhines, including *Callicebus* and all three pitheciine genera. Their data show that the ratio of the temporalis and superficial part of the masseter was similarly high in *Cacajao* (2.30) and *Chiropotes* (2.14), as compared with the lower values in *Pithecia* (1.70) and *Callicebus* (1.60). However, the PCSA values reported by Taylor *et al*. [79] for pitheciids differ notably from those published in earlier studies. In particular, the PCSA values for the jaw adductors in *Callicebus* are around five times greater than those in Anapol *et al*. [75]. Additionally, *Chiropotes* and *Pithecia* have much more similarly-sized muscles in the Taylor *et al*. [79] study, with smaller values reported for *Chiropotes* and larger values for *Pithecia*. These discrepancies are likely the result of age-related muscle loss in the captive animals that were included in their sample, which the authors note with reference to their pitheciid sample, although it is unclear which specimens were affected by this. For these reasons, and because Taylor *et al*. [79] did not include deep masseter or medial pterygoid in their analysis, we did not incorporate these data into our study.

For all analyses, groups of plate elements representing each muscle’s origin (Fig 2B and 2C) were created by tessellating the surface faces of tet4 elements and modeling them as 3D membrane (thickness = 0.0001 mm; *E* = 17.3 GPa; *v* = 0.28). These muscle origins were based on photographs taken during dissections of the *Chiropotes* specimen discussed above and four other specimens housed at Stony Brook University (two specimens each of *Pithecia* and *Chiropotes*), as well as published muscle maps [80]. Using Boneload [81], forces for each muscle plate group were then applied as tractions directed toward their respective insertions on the mandible. Insertion sites were defined using focal coordinates corresponding to the centroid of each muscles’ insertion area, with the mandibles of all models set to the same gape angle of 12° between the tips of the P^3^ cusps (see Fig 2C). Adducting the mandible to this degree provided enough clearance for the canines in pitheciines so that they could be used in sclerocarpy, with a need for large gapes lessened by the lateral splay of the canines. This angle is also smaller than but near to the maximum gape angles used by *Callicebus* and *Pithecia* during ingestion in captive conditions [82]. During muscle loading, the models were oriented to a line best fit to the postcanine occlusal plane [83].

Models were subjected to separate trials of unilateral canine biting by constraining a single node from vertical displacement at the left canine tip. Molar biting was analyzed by constraining a single node in the center of the left M^2^. During each load case, a single node was also constrained against translation in all directions at the working- and balancing-side temporomandibular joint (TMJ), using the position of the condyles as a guide. These constraints create an axis of rotation around the TMJs, inducing deformation in the craniofacial skeleton and generating reaction forces at the constrained nodes upon the application of muscle forces.

### Analysis of model output parameters

We compared mechanical performance in our FEMs using data on strain magnitudes and bite force leverage. Global von Mises strains were examined using color-coded strain maps, which provide information on both strain magnitude and the spatial patterning of strain concentrations. Strain data were also collected from each model at 20 sites across the craniofacial skeleton (see below). These locations include those used in previous analyses of primate feeding biomechanics (e.g., [56, 57, 84, 85]). Data on reaction forces generated at the constrained teeth and two TMJs were recorded in Newtons (N). Reaction forces at the left canine and M^2^ were recorded relative to the occlusal plane, while reaction forces at the left and right jaw joints were recorded and compared relative to a user-defined “triangle of support” Cartesian coordinate system, with one of three axes perpendicular to a reference plane defined by the “triangle of support” formed by the constrained nodes at the bite point and two articular eminences [7]. The efficiency of bite force production at a given bite point in each model was also compared using the mechanical advantage (MA), a measure of leverage, calculated as the ratio of bite force (output) to muscle force (input).

## Results

**Fig 3:**
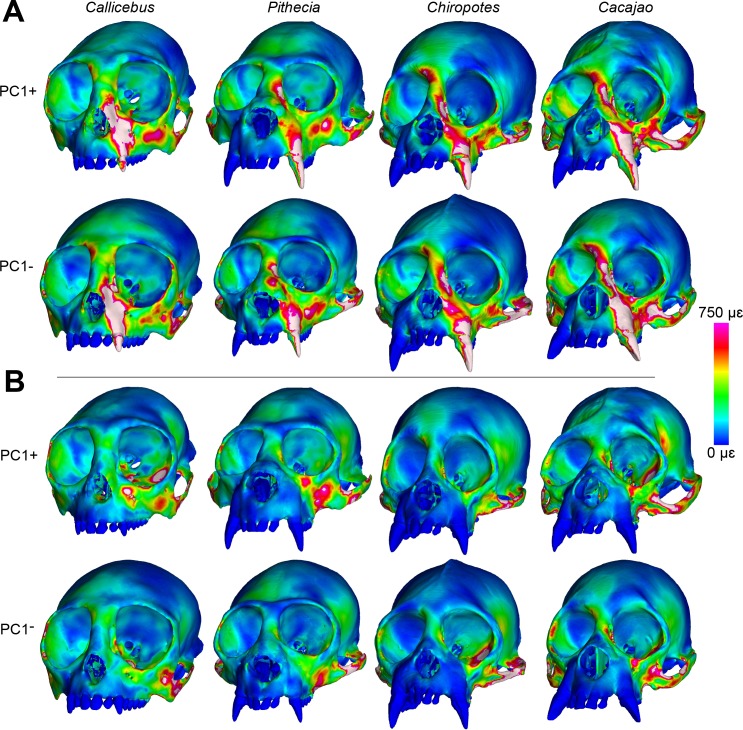
Color mapping of Batch 1 strain results. Color maps of von Mises microstrain (με) distributions in FEMs of *Callicebus*, *Pithecia*, *Chiropotes*, and *Cacajao* using Batch 1 muscle forces (without species-specific force ratios) for (**A**) canine and (**B**) M^2^ biting. “Cool” colors represent areas of low strain, whereas “warm” colors indicate larger strain magnitudes. White regions exceed 750 με. Models are shown at roughly the same facial height (i.e., not to scale) to accentuate similarities and differences in strain distribution.

Color maps of von Mises microstrain during the first set of canine and M^2^ analyses (Batch 1, without species-specific muscle force ratios) are shown in Fig 3. During canine biting, large strain were generated along the working-side (left) nasal margin and infraorbital region of *Callicebus*. Strains are lower in the sakis and bearded sakis, but the uakaris experienced large strain magnitudes in the zygomatic and interorbital regions, as well as high strains along the nasal margin, corresponding to the canine root. Differences between taxa were less pronounced for M^2^ biting, but the color maps indicate that strains were generally lowest in *Chiropotes*. *Callicebus* exhibits elevated strain concentrations in the working zygoma and at the medial infraorbital, but high strain magnitudes also occur in *Cacajao* surrounding the intersection of the temporal and frontal processes of the zygomatic (i.e., the jugal region), as well as in *Pithecia* near the lateral infraorbital superior to the working zygomatic root.

**Fig 4:**
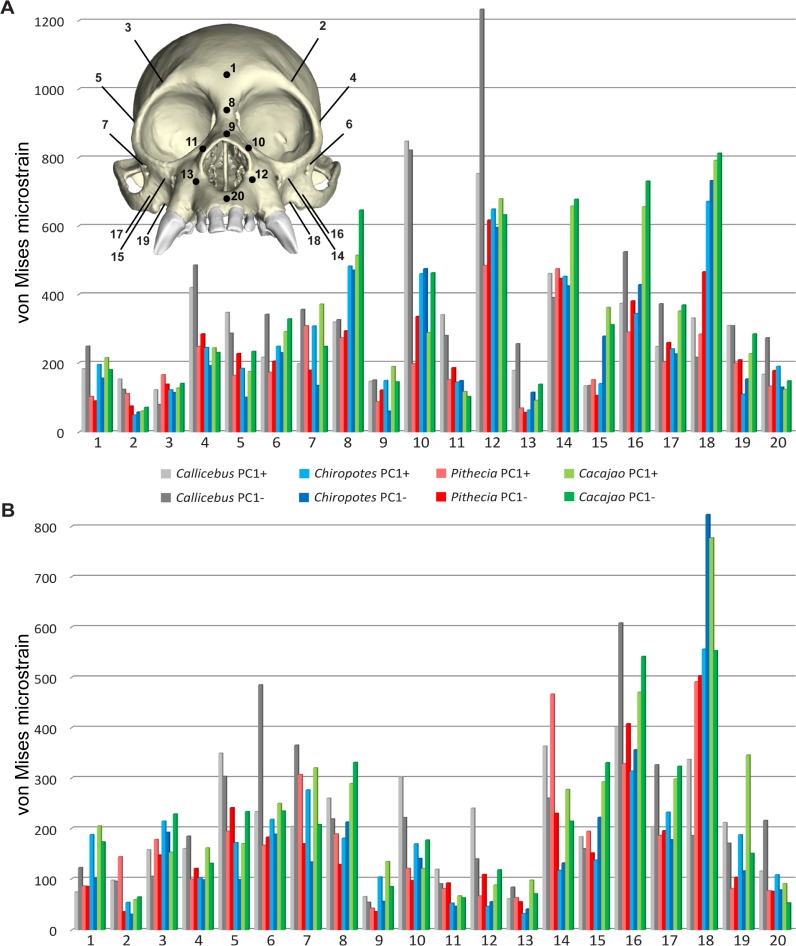
Plot of Batch 1 strain data from sampled regions. The von Mises microstrain (με) magnitudes sampled from 20 craniofacial sites in FEMs of *Callicebus*, *Pithecia*, *Chiropotes*, and *Cacajao* for Batch 1 during (**A**) canine and (**B**) M^2^ biting.

These results are supported by von Mises microstrain data collected from 20 craniofacial sites (Fig 4, Table 5). Of these 20 locations, peak strains during canine biting were highest in *Callicebus* at 12, with most of these well above and not overlapping with the ranges of other taxa. The working nasal margin of *Callicebus* (sites 10 and 12) experienced particularly high strain magnitudes. *Cacajao* experienced the highest strain at seven sites, notably the working infraorbital (site 14) and zygoma (site 16). *Pithecia* experienced the highest strain at only one site, the balancing-side dorsal orbital (site 3). However, strains were overall lowest in *Pithecia* (lowest at nine sites), followed by *Chiropotes* (lowest at six sites) during the canine bite. Only site 11 followed the predicted *Callicebus*-*Pithecia*-*Chiropotes*-*Cacajao* morphocline of increasing stiffness (i.e., decreasing strain), whereas site 18 experienced a reverse trend and 8 others (sites 1, 6, 8, 12, 13, 15, 16, 18) show a reverse trend within pitheciines (*Cacajao*-*Chiropotes*-*Pithecia*). For M^2^ biting, strain magnitudes were greatest in *Callicebus* at 10 sites, while *Cacajao* generated the highest strain at 7 sites. Although *Chiropotes* and *Pithecia* exhibited high stiffness across much of the face, three sites (sites 2, 14, 18) experienced high strain magnitudes. Overall, strain was lowest in *Chiropotes* during this load case.

**Table 5:** Batch 1 strain data from sampled regions. The von Mises microstrain (με) magnitudes sampled from 20 craniofacial sites in FEMs of *Callicebus*, *Pithecia*, *Chiropotes*, and *Cacajao* during canine and molar biting, without species-specific muscle force ratios (Batch 1).

Bite	Site	*Callicebus* PC1+	*Callicebus* PC1-	*Pithecia* PC1+	*Pithecia* PC1-	*Chiropotes* PC1+	*Chiropotes* PC1-	*Cacajao* PC1+	*Cacajao* PC1-
Canine	1	182	248	102	89	194	155	215	179
	2	152	123	111	75	49	58	60	71
	3	121	79	165	137	122	113	126	140
	4	419	485	247	284	244	191	243	229
	5	347	286	163	227	183	100	174	233
	6	216	341	172	205	248	229	290	328
	7	197	356	308	178	307	134	371	247
	8	319	325	272	293	482	470	514	646
	9	146	149	87	121	148	60	189	144
	10	847	821	197	335	460	475	288	462
	11	340	279	151	185	143	148	116	102
	12	752	1233	484	617	649	595	679	632
	13	177	255	69	56	63	114	91	137
	14	460	390	475	446	452	424	657	677
	15	133	134	150	105	139	277	361	311
	16	373	524	289	380	344	428	655	731
	17	247	372	202	259	240	225	351	369
	18	330	215	283	466	671	732	791	812
	19	308	308	198	208	109	152	226	284
	20	166	273	132	177	189	129	123	147
M^2^	1	74	122	86	85	187	101	205	173
	2	97	95	143	35	54	30	59	64
	3	157	105	178	147	214	192	152	228
	4	159	184	99	120	101	98	161	130
	5	349	303	194	241	171	98	169	233
	6	233	485	166	182	217	188	249	234
	7	204	365	307	169	276	133	320	207
	8	260	219	188	128	180	212	289	331
	9	65	54	42	36	103	56	134	84
	10	302	221	120	96	169	140	120	176
	11	118	90	80	92	52	46	66	63
	12	240	139	66	108	46	55	87	117
	13	61	83	63	55	32	41	97	70
	14	363	260	466	230	116	131	277	214
	15	183	159	194	151	137	222	292	330
	16	402	608	328	408	314	356	470	541
	17	203	326	186	195	232	177	298	323
	18	337	185	491	503	556	823	777	553
	19	211	170	80	103	187	115	345	150
	20	115	216	76	75	108	78	90	53

Data on bite force production and joint reaction forces are shown in Table 6. When not accounting for species-specific muscle force ratios (Batch 1), we found that the mechanical efficiency of canine biting increased from *Callicebus* to *Pithecia* to *Chiropotes*, but *Cacajao* exhibited the same efficiency as *Chiropotes*. A similar pattern describes second molar biting, except with higher MA in *Chiropotes* than *Cacajao*. As predicted by the constrained lever model of feeding biomechanics [86, 87], canine biting generated strongly compressive reaction forces at both jaw joints. However, both models of *Chiropotes* and *Cacajao*, and to a much lesser extent one *Pithecia* model (PC1+), experienced distractive (tensile) reaction forces at the working jaw joint when biting at the molar.

**Table 6:** Bite force production. Bite force (BF), mechanical advantage (MA), working-side TMJ reaction force (WJRF), and balancing-side TMJ reaction force (BJRF) for canine and molar (M^2^) biting. All forces are in Newtons (N), with positive values indicating compression and negative values indicating tension. The MA, a measure of biting leverage or efficiency, was calculated as the ratio of BF to total input muscle force.

			Canine Bite				Molar Bite			
Model	Muscle Force^1^	Batch	BF	MA	WJRF	BJRF	BF	MA	WJRF	BJRF
*Callicebus* PC1+	162.21	1	42.29	0.26	26.88	41.01	73.94	0.46	3.04	37.34
		2	44.44	0.27	26.52	41.65	77.73	0.48	1.99	38.34
*Callicebus* PC1-	165.97	1	38.41	0.23	32.74	44.88	69.37	0.42	7.58	40.01
		2	40.47	0.24	32.24	45.31	73.09	0.44	6.29	40.76
*Pithecia* PC1+	253.77	1	72.84	0.29	22.92	64.22	124.12	0.49	-8.14	47.51
		2	77.19	0.30	28.25	71.63	131.54	0.52	-3.98	54.61
*Pithecia* PC1-	226.73	1	63.03	0.28	33.89	64.89	108.78	0.48	3.73	51.68
		2	66.64	0.29	36.89	69.49	115.01	0.51	5.72	56.21
*Chiropotes* PC1+	415.64	1	128.56	0.31	38.44	122.25	242.69	0.58	-30.11	81.64
*Chiropotes* PC1-	472.80	1	161.39	0.34	23.87	115.80	284.55	0.60	-44.49	73.30
*Cacajao* PC1+	499.17	1	171.56	0.34	19.56	122.86	285.03	0.57	-37.17	78.09
		2	166.22	0.33	15.65	115.42	276.15	0.55	-39.34	72.02
*Cacajao* PC1-	503.01	1	155.54	0.31	49.88	155.55	275.26	0.55	-12.21	100.95
		2	148.67	0.30	49.77	150.08	263.11	0.52	-11.11	96.24

^1^Bilateral muscle force.

**Fig 5:**
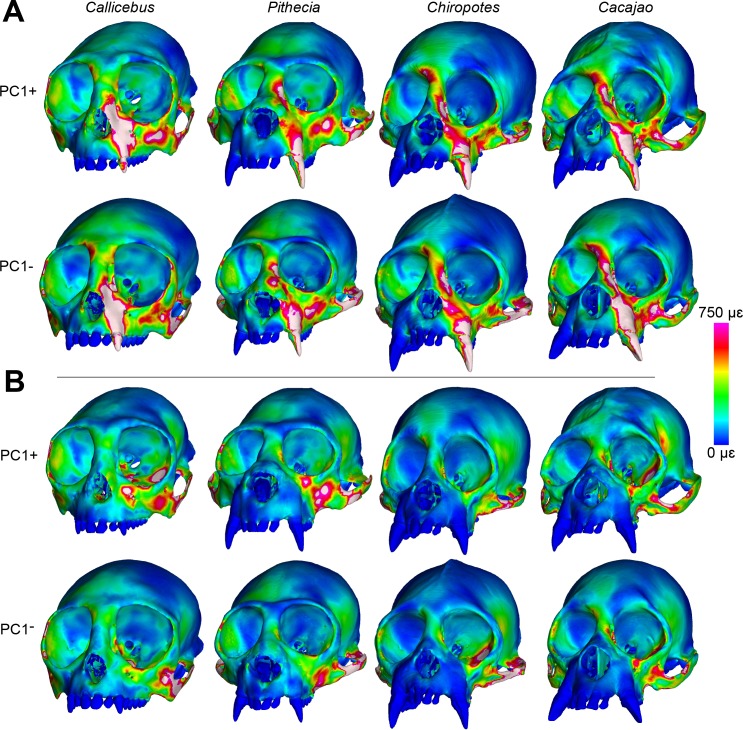
Color mapping of Batch 2 strain results. Color maps of von Mises microstrain (με) distributions in FEMs of *Callicebus*, *Pithecia*, *Chiropotes*, and *Cacajao* using Batch 2 muscle forces (including species-specific force ratios) for (**A**) canine and (**B**) M^2^ biting. “Cool” colors represent areas of low strain, whereas “warm” colors indicate larger strain magnitudes. White regions exceed 750 με. Models are shown at roughly the same facial height (i.e., not to scale) to accentuate similarities and differences in strain distribution.

The redistribution of temporalis and masseter muscle forces in *Callicebus*, *Pithecia*, and *Cacajao* according to species-specific muscle force ratios (Batch 2) had a noticeable effect on the relative differences between models, but did not drastically alter the pattern observed in the Batch 1 results. In both *Callicebus* and *Pithecia*, strain was found to increase during both canine and M^2^ biting, particularly in the zygoma and infraorbital regions, whereas strain in these areas experienced a slight decrease in *Cacajao* (Fig 5, Table 7). Of the 20 locations sampled, strains during canine biting were greatest in *Callicebus* at 13, with most of these well above and not overlapping with the ranges of other taxa. *Cacajao* experienced the highest strain at six sites, whereas *Pithecia* experienced the highest strain at only one site. Strains were overall lowest in *Chiropotes* (lowest at nine sites) and *Pithecia* (lowest at seven sites). As with the Batch 1 results, only site 11 followed the predicted *Callicebus*-*Pithecia*-*Chiropotes*-*Cacajao* trend of increasing stiffness (decreasing strain). Site 18 experienced a reverse trend and five others show a reverse pattern within pitheciines (*Cacajao*-*Chiropotes*-*Pithecia*). During M^2^ biting, strain magnitudes were greatest in *Callicebus* at 12 sites, while *Cacajao* generated the highest strain at five sites. Although *Chiropotes* and *Pithecia* once again exhibited high stiffness across much of the face, three sites (sites 3, 14, 18) experienced high strain magnitudes. Overall, strain was lowest in *Chiropotes* during this load case. With respect to bite force, both canine and M^2^ MA were found to increase in *Callicebus* and *Pithecia*, whereas MA slightly decreased in *Cacajao* when applying species-specific muscle force ratio data (see Table 6).

**Table 7:** Batch 2 strain data from sampled regions. The von Mises microstrain (με) magnitudes sampled from 20 craniofacial sites in FEMs of *Callicebus*, *Pithecia*, *Chiropotes*, and *Cacajao* during canine and molar biting, including species-specific muscle force ratios (Batch 2).

Bite	Site	*Callicebus* PC1+	*Callicebus* PC1-	*Pithecia* PC1+	*Pithecia* PC1-	*Chiropotes* PC1+	*Chiropotes* PC1-	*Cacajao* PC1+	*Cacajao* PC1-
Canine	1	183	262	97	87	194	155	223	183
	2	173	150	95	92	49	58	63	88
	3	139	90	203	159	122	113	108	119
	4	474	565	290	323	244	191	232	213
	5	403	339	200	271	183	100	147	196
	6	241	411	214	232	248	229	304	346
	7	241	441	400	224	307	134	313	206
	8	317	335	280	301	482	470	517	634
	9	163	163	96	132	148	60	170	133
	10	901	909	224	370	460	475	270	425
	11	378	313	178	210	143	148	93	79
	12	803	1310	526	663	649	595	644	593
	13	198	276	83	67	63	114	75	129
	14	513	426	530	486	452	424	592	626
	15	159	156	194	131	139	277	306	255
	16	439	619	327	403	344	428	601	666
	17	296	447	260	316	240	225	292	308
	18	379	250	294	483	671	732	702	757
	19	354	343	208	232	109	152	155	305
	20	191	295	139	189	189	129	125	144
M^2^	1	75	132	78	80	187	101	212	176
	2	117	120	117	28	54	30	44	72
	3	180	125	217	168	214	192	132	203
	4	192	246	126	138	101	98	163	130
	5	406	357	232	286	171	98	143	197
	6	265	554	164	196	217	188	266	254
	7	249	451	398	216	276	133	263	166
	8	273	227	198	130	180	212	287	328
	9	81	62	51	42	103	56	117	76
	10	330	245	143	115	169	140	108	148
	11	148	116	103	113	52	46	47	43
	12	262	156	68	124	46	55	78	101
	13	77	98	78	67	32	41	80	55
	14	395	270	528	254	116	131	238	193
	15	210	181	236	177	137	222	236	273
	16	463	706	364	435	314	356	425	486
	17	251	399	243	251	232	177	240	263
	18	391	219	517	531	556	823	688	507
	19	253	200	101	136	187	115	258	110
	20	142	259	87	87	108	78	76	46

## Discussion

We analyzed feeding biomechanics in pitheciine monkeys (*Pithecia*, *Chiropotes*, *Cacajao*), a clade that specializes on hard-husked unripe fruit (sclerocarpy) and mechanically resistant seeds (seed predation). We tested the hypothesis (Hypothesis 1) that pitheciine crania are well-suited to *generate* and *withstand* forceful canine and molar biting, with the prediction that pitheciine species would generate bite forces more efficiently and would better resist masticatory strains relative to the closely-related *Callicebus*, which does not specialize on unripe fruits and/or seeds. We also tested the hypothesis (Hypothesis 2) that *Callicebus*-*Pithecia*-*Chiropotes*-*Cacajao* represent a morphocline of increasing sclerocarpic specialization with respect to bite force leverage and craniofacial strength, consistent with Kinzey’s [10] proposed morphocline of anterior dental specialization.

We found that pitheciines generate bite forces more efficiently than *Callicebus*, supporting the predictions of Hypothesis 1. All three pitheciine taxa, particularly *Chiropotes* and *Cacajao*, exhibited elevated mechanical advantage at both the canine and second molar bite points. This finding agrees with earlier studies of pitheciine feeding biomechanics using two-dimensional (2D) estimates of leverage and load arms [21, 31, 48], providing further evidence for the relationship between diet and bite force efficiency in primates that exploit foods that are hard or difficult to crack open (see also [88, 89]).

We found that pitheciines experienced generally lower von Mises microstrain magnitudes than *Callicebus*, with the possible exception of *Cacajao*. During both biting simulations, *Pithecia* and *Chiropotes* generated the lowest strain at almost every sampled region. However, uakaris experienced the highest strain magnitudes at several craniofacial sites examined during both canine and molar biting, making them second to *Callicebus* in terms of craniofacial “weakness”. Further, examination of von Mises strain during canine biting in *Cacajao* and *Callicebus* (see Fig 3) reveals regions of higher strain in the former around the jugal region and the interorbital/glabellar region. Therefore, *Cacajao* is arguably less strong than *Callicebus* depending on the region being compared. These results provide mixed support for Hypothesis 1.

The relative differences between models did not change much with the inclusion of species-specific muscle force ratio data (i.e., Batch 2). However, bite force leverage and strain did increase to a small degree in both *Callicebus* and *Pithecia*, while these metrics were both found to decrease slightly in *Cacajao* (see Fig 5 and Tables 6 and 7). These changes are most apparent in the zygoma, particularly the superior root of the zygomatic arch, and infraorbital regions. In *Callicebus* and *Pithecia*, the observed changes reflect a decrease in the temporalis force and increase in the masseter muscle force, whereas forces in *Cacajao* were redistributed to have higher temporalis and lower masseter forces. These results support previous studies suggesting that inferior bending of the zygomatic arch under the pull of the masseter muscle force and the resulting frontal shearing of the infraorbital and zygoma are predominant loading regimes in primates [56], including humans [67, 90, 91]). The arch is potentially stabilized by the tensile strength of the temporalis fascia [92], which inserts onto its superior border. However, previous FEA studies of primate feeding that did not include the temporalis fascia (e.g., [56, 93–95] generated strains more similar in magnitude to those collected during *in vivo* bone strain experiments [84, 85, 96]. Therefore, we did not feel that it was necessary to include this structure in our FEMs.

Given the results discussed above, we find limited support for of Hypothesis 2, that mechanical performance would follow Kinzey’s [10] proposed *Callicebus*-*Pithecia*-*Chiropotes*-*Cacajao* morphocline of sclerocarpic specialization, developed on the basis of anterior dental specialization. Consistent with our muscle force scaling approach, absolute bite forces increased with increasing model size, a trend that follows the predicted morphocline (see Table 5). Size alone may therefore allow uakaris to access the hardest-husked fruits, although Taylor *et al*. [79] report PCSA values for *Cacajao* that are very similar to the data for *Chiropotes* published by Anapol *et al*. [75] (Taylor and colleagues report lower values for *Chiropotes*). However, as discussed above, *Cacajao* was found to exhibit canine biting leverage more or less identical to that in *Chiropotes* when not accounting for species-specific muscle force ratios (Batch 1), with our results supporting a *Callicebus*-*Pithecia*-*Chiropotes*/*Cacajao* gradient of increasing canine MA. When muscle forces were redistributed according to species-specific jaw adductor force ratios (Batch 2), canine and molar biting efficiency in *Cacajao* decreased and were found to overlap with *Pithecia*. With respect to strain magnitudes during canine biting, only one site (site 11) followed the expected trend for both Batch 1 and Batch 2. *Cacajao* was far from the strongest taxon examined, if not the weakest. Although *Callicebus* was weaker at more than half of the 20 sites examined, only five (Batch 1) or six (Batch 2) sites exhibited a *Callicebus*-*Pithecia*-*Chiropotes* trend of increasing stiffness. In fact, strains at the sample locations were overall lower in *Pithecia* than in *Chiropotes* during canine biting, in contrast to the proposed morphocline. This is further evidenced by comparing the average von Mises strains during canine biting from all 20 craniofacial sites using the data in Table 5. This comparison masks large differences in strain at particular facial sites (e.g., high strain at the nasal margin, site 12, of *Callicebus*), but nonetheless it reveals that *Pithecia* experienced the lowest average strain for both Batch 1 (227 με) and Batch 2 (255 με). However, *Chiropotes* experienced only slightly higher average strain magnitudes (266 με for both Batches). The average von Mises strain data also further demonstrate that the most highly strained taxa in our sample were *Callicebus* and *Cacajao*, which experienced remarkably similar average strain magnitudes of 336 με and 335 με, respectively, during Batch 1. The difference between these taxa was greater during Batch 2, with *Callicebus* experiencing a higher average strain value (376 με) than *Cacajao* (308 με).

Interestingly, a greater number of sites show a pattern within pitheciines (*Cacajao*-*Chiropotes*-*Pithecia*) during canine biting (8 for Batch 1 or 5 for Batch 2) that is the reverse of the predicted trend. This could indicate that increases in canine bite force leverage and enhancements in anterior dental specialization as proposed by Kinzey [10], including larger and more laterally splaying canines and procumbent incisors, were selected for at the expense of increased strain magnitudes in some regions of the face. These results provide some support for the conclusions of Ross and Iriarte-Diaz [49], who note that primate craniomandibular morphology “reflects trade-offs in primate feeding system design enforced by the multiple performance criteria that the feeding system must meet” (pg. 117). We suggest that pitheciine feeding biomechanics and dietary adaptation represent a functional compromise between anterior dental specialization, increased bite force leverage, and maintaining an appropriate safety factor with respect to bone strain and structural strength. Adaptations for increasing bite force leverage have also been shown to increase craniofacial strain magnitudes in australopiths, and certain facial buttressing features may have evolved to compensate for this reduced structural stiffness [52]. However, in pitheciines, elevated strains resulting from specialized sclerocarpy remain low relative to the strain magnitudes experienced by larger species (e.g., chimpanzees [57]), so may not require additional bony adaptations to mitigate their weakening effect.

Although the trend of increasingly more specialized anterior teeth in pitheciines might explain our strain results, these adaptations are unlikely to explain some of the unexpectedly high strains observed in our *Cacajao* FEMs. Notably, the zygomaticoalveolar crest (ZAC) of *Cacajao* is more curved than in either saki. A straighter and more steeply-inclined ZAC is thought to better resist masticatory stresses associated with masseteric contraction and the bite reaction force [1, 52]. The more curved ZAC of *Cacajao* could therefore explain high strain magnitudes at the superior and inferior roots of the zygomatic arch. This potentially reflects a reduction in the need for regular de-husking of unripe fruits and reduced selection for maintaining robust facial features in *Cacajao*, perhaps offered by allopatry and relaxed feeding competition with other pitheciine species. However, it should be noted that *Chiropotes* also exhibited high strains at the inferior zygomatic root (site 18) during molar biting.

Uakaris are not broadly sympatric with either saki, so may not be limited to eating the hardest-husked fruits. Compared with *Pithecia* and *Chiropotes*, less work has been done on the mechanical properties of foods in the diets of *Cacajao* species, but many studies note that uakaris prefer fruits with hard husks (e.g.,[27, 28]). Indeed, Ayres [25] notes that the preference for hard fruits in both *Chiropotes satanas* and *Cacajao calvus* are likely to explain their mutual avoidance of each other. However, Barnett *et al*. [44] found that golden-backed uakaris at Jau National Park (Brazil) often feed on softer fruits when available. This suggests that the relative lack of competition may allow uakaris to choose from a wider variety of fruits and seeds at various stages of ripeness. Indeed, low occlusal complexity in *Cacajao* relative to other pitheciines might also reflect relaxed dietary resource competition, specifically a reduced reliance on leaves as a source of dietary fiber and/or greater access to fruit seeds with lower fracture toughness [22]. This form of competitive exclusion is strongest among primate taxa that exploit less readily available and less ubiquitous resources [97, 98], such as unripe fruit seeds. Competition remains high between sympatric *Pithecia* and *Chiropotes*, driving dietary niche separation in these taxa characterized by intensified sclerocarpy in the latter [9, 26, 32], whereas *Cacajao* occupies a nearly competition-free ecological niche [43].

Although the craniofacial skeleton of *Cacajao* may be not be remarkably stiff, Norconk *et al*. [31] found that the mandible of *Ca*. *melanocephalus* was the second most “robust” species included in their platyrrhine sample, occupying an intermediate position between the slightly stronger *Chiropotes satanas* and slightly weaker *Cebus* (*Sapajus*) *apella*. Other studies find the mandible of *Cacajao* to be similarly robust [20]. Therefore, a hypothesis that has yet to be tested fully is that the mandible is more highly influenced by selection for increased strength in pitheciines, and perhaps across all primates [68]. Indeed, although cranial shape reflects a compromise between numerous integrated functions (e.g, cognition, respiration, feeding), perhaps masking a strong dietary signal in some primate groups (e.g., lemurs [99]), the mandible may be more closely linked to diet and feeding behavior, although evidence for this relationship also appears to be weak [49, 100].

In addition to comparisons of the puncture resistance of fruits breached by *Pithecia* and *Chiropotes*, Norconk and colleagues [9, 26, 32] compared the crushing resistance of seeds eaten by these taxa and found that seeds masticated by *Pithecia* have significantly higher crushing resistance. In contrast to these ecological data, we found that *Pithecia* generated less efficient molar bite forces and experienced higher von Mises strain magnitudes than *Chiropotes*. This was in addition to *Pithecia* generating absolutely lower bite forces. However, unlike *Pithecia*, *Chiropotes* generated highly distractive (tensile) joint reaction forces at the working-side TMJ during molar biting (see Table 5). These distractive forces “pull” the working-side mandibular condyle from the articular eminence, increasing the risk of damaging the soft tissues of the joint capsule when biting forcefully on the back teeth [86, 87]. Therefore, although *Chiropotes* is capable of generating high bite forces more efficiently at its distal molars than *Pithecia*, it is likely that they reduce tensile loading of the jaw joint by avoiding powerful molar biting behaviors. Instead, *Chiropotes* is more suited for the efficient grinding of softer seeds (from harder fruits), as suggested by the published crushing resistance data. In this scenario, high molar bite force efficiency is viewed as a byproduct of strong selection for increased anterior bite force efficiency.

Evidence in support of this hypothesis comes from Spencer’s [47] study of pitheciine postcanine tooth root morphology. He found that, unlike *Pithecia*, *Chiropotes* exhibits a decrease in the number of molar tooth roots and a striking decrease in molar tooth root surface area in a gradient from M^1^ to M^3^, suggesting that this finding reflects a decreased reliance on forceful biting at the distal molars. Ledogar [101] similarly found a significantly smaller M^3^ occlusal surface area in *Chiropotes* than *Pithecia* when scaled to a geometric mean of skull size. Consistent with this observation, several studies have linked increased jaw adductor leverage with third molar reduction and agenesis [47, 48, 102]. With an anterior shift of the muscles, the third molar may fall within Region III of the tooth row [102]. Forceful biting in this region increases the chances of TMJ distraction by shifting the muscle resultant vector outside of the triangle of support formed by the mandibular condyles and the bite point [86]. Thus, the functional area of the postcanine tooth row is reduced and the expected result is a reduction of molar occlusal surface area, particularly the third molar, or even agenesis of this tooth. Third molar agenesis is common in modern humans [103], which has been linked to the combination of high bite force leverage and TMJ distraction found to also characterize human molar biting [67]. However, human bite force efficiency is the result of facial retraction, as opposed to an anterior migration of the masticatory muscles, and there are various non-masticatory hypotheses purporting to explain the evolution of facial flatness in humans [104].

## Conclusion

We found that the feeding biomechanics of pitheciine monkeys (*Pithecia*, *Chiropotes*, *Cacajao*) are consistent with a diet that includes mechanically resistant foods (unripe fruits and seeds). During simulations of canine and second molar biting, pitheciines out-performed *Callicebus* with respect to the mechanical efficiency (leverage) of bite force production and resistance to masticatory strain. However, *Cacajao* was found to experience unexpectedly high facial strain magnitudes relative to the other pitheciines in some regions of the face. We therefore found limited support for the hypothesis that *Callicebus*-*Pithecia*-*Chiropotes*-*Cacajao* represent a morphocline of increasing sclerocarpic specialization [10], at least with respect to the mechanical performance metrics examined here. In fact, strain magnitudes during canine biting were more likely to follow a *Cacajao*-*Chiropotes*-*Pithecia* trend of increasing craniofacial strength, in contrast to the proposed morphocline. On the other hand, we found that *Callicebus*-*Pithecia*-*Chiropotes*/*Cacajao* represent a gradient of increasing canine MA, with leverage in *Cacajao* nearly identical to (or slightly less than) in *Chiropotes*. Together, these results could indicate that increased bite force efficiency and increasingly derived anterior dental morphology was selected for in pitheciines at the expense of higher craniofacial strain magnitudes. However, the unexpectedly weak facial skeleton of *Cacajao* potentially reflects reduced feeding competition with other pitheciines; this reduced competition allows uakaris to choose from a wider variety of fruits at various stages of ripeness, leading to reduction in the selection for robust facial features.

We also found that *Pithecia* and *Chiropotes* exhibit differences in biting performance that are consistent with food structural property data and previous observations of dietary niche separation between these sister taxa [9, 26, 32], supporting the hypothesis that sakis and bearded sakis are able to reduce competition and remain sympatric by specializing on fruits and seeds at different stages of ripeness. Consistent with data on fruit puncture resistance, we found that bite force production and efficiency during canine biting was greatest in *Chiropotes*. Biting leverage was also great at the molar in *Chiropotes*, but *Pithecia* was found to be better suited for the regular forceful crushing and grinding of seeds using the postcanine teeth, consistent with data on seed crushing resistance and molar root surface area.

Future studies investigating the dietary ecology of pitheciines should focus on the collection of mechanical properties of foods eaten by *Cacajao*. Although uakaris have traditionally been considered a flooded forest specialist (e.g., [42]), which has complicated attempts to study their dietary ecology, recent work indicates that not all species are restricted to such habitats. A recent review of all available information concerning the habitat of *Ca*. *calvus ucayalii* [105] concluded that this species is more flexible in habitat preference than previously thought and most frequently occupies non-flooded terra firme forests or mixed habitats. For example, uakaris at the Lago Preto Conservation Concession in Iquitos, Peru are commonly associated with terra firme forest [106], which offers high potential for a detailed study of *Cacajao* dietary ecology and the collection of food mechanical properties. Future work on feeding ecology in *Cacajao* and other pitheciines will also allow for more detailed collection of data on fruit size, gape, and paramasticatory feeding behaviors, such as when using the hands to pull fruits or when manipulating a fruit to avoid biting along its largest dimension [31]. One limitation of the current study is that our biting simulations have not taken the broad range of such behaviors into account.

Much-needed research exploring the relationship between primate food mechanical/structural properties and their ecological significance has been growing in recent years (e.g., [31, 36, 48, 107–117]). With respect to pitheciines, Norconk and colleagues [9, 26, 32] quantify fruit and seeds eaten by *Pithecia* and *Chiropotes* in terms of their puncture and crushing resistance (i.e., structural properties). Lucas [118] suggests that the mechanical properties *E* (Young’s modulus, a measure of material stiffness) and *R* (fracture toughness, a measure of resistance to fracture propagation) more accurately describe food fragmentation during ingestion and mastication, and may have more of a direct influence on the evolution of the masticatory apparatus. Therefore, it is an appropriate question as to whether *E* and *R* explain niche partitioning in pitheciines equally well. Future work should focus on the incorporation of both structural and mechanical food properties into the analysis of craniofacial form in pitheciines.

## References

[1] Rak Y. The australopithecine face New York: Academic Press; 1983.

[2] Strait DS, Weber GW, Neubauer S, Chalk J, Richmond BG, Lucas PW, et al The feeding biomechanics and dietary ecology of *Australopithecus africanus*. P Natl Acad Sci USA. 2009;106: 2124–2129. 10.1073/pnas.0808730106 PMC2650119 19188607

[3] Perry JMG, Kay RF, Vizcaino SF, Bargo MS. Tooth root size, chewing muscle leverage, and the biology of *Homunculus patagonicus* (Primates) from the late early Miocene of Patagonia. Ameghiniana. 2010;47: 355–371.

[4] Dumont ER, Ryan TM, Godfrey LR. The *Hadropithecus* conundrum reconsidered, with implications for interpreting diet in fossil hominins. P Roy Soc B-Biol Sci. 2011;278: 3654–3661. 10.1098/rspb.2011.0528 PMC3203504 21525060

[5] Perry JMG, St Clair EM, Hartstone-Rose A. Craniomandibular signals of diet in adapids. Am J Phys Anthropol. 2015;158: 646–662. doi: 10.1002/ajpa.22811 26174869 10.1002/ajpa.22811

[6] Strait DS, Constantino P, Lucas PW, Richmond BG, Spencer MA, Dechow PC, et al Viewpoints: Diet and dietary adaptations in early hominins: The hard food perspective. Am J Phys Anthropol. 2013;151: 339–355. doi: 10.1002/ajpa.22285 23794330 10.1002/ajpa.22285

[7] Smith AL, Benazzi S, Ledogar JA, Tamvada K, Smith LCP, Weber GW, et al The feeding biomechanics and dietary ecology of *Paranthropus boisei*. Anat Rec. 2015;298: 145–167. 10.1002/ar.23073 PMC4420635 25529240

[8] Kay RF, Cartmill M. Cranial morphology and adaptations of *Palaechthon nacimienti* and other paromomyidae (Plesiadapoidea: Primates), with description of a new genus and species. J Hum Evol. 1977;6: 19–53.

[9] Kinzey WG, Norconk MA. Hardness as a basis of fruit choice in two sympatric primates. Am J Phys Anthropol. 1990;81: 5–15. doi: 10.1002/ajpa.1330810103 2301558 10.1002/ajpa.1330810103

[10] Kinzey WG. Dietary and dental adaptations in the Pitheciinae. Am J Phys Anthropol. 1992;88: 499–514. doi: 10.1002/ajpa.1330880406 1503121 10.1002/ajpa.1330880406

[11] Schneider H, Canavez FC, Sampaio I, Moreira MAM, Tagliaro CH, Seuanez HN. Can molecular data place each Neotropical monkey on its own branch? Chromosoma. 2001;109: 515–523. 11305784 10.1007/s004120000106

[12] Finotelo LFM, Amaral PJS, Pieczarka JC, de Oliveira EHC, Pissinati A, Neusser M, et al Chromosome phylogeny of the subfamily Pitheciinae (Platyrrhini, Primates) by classic cytogenetics and chromosome painting. Bmc Evol Biol. 2010;10. 10.1186/1471-2148-10-189 PMC2905426 20565908

[13] Ford SM. Systematics of the New World monkeys In: Swindler DR, Erwin J, editors. Comparative primate biology, Vol 1, Systematics, evolution, and anatomy. New York: Alan R Liss, Inc; 1986 pp. 73–135.

[14] Kay RF. The phyletic relationships of extant and fossil Pitheciinae (Platyrrhini, Anthropoidea). J Hum Evol. 1990;19: 175–208.

[15] von Dornum M, Ruvolo M. Phylogenetic relationships of the New World monkeys (Primates, Platyrrhini) based on nuclear G6PD DNA sequences. Mol Phylogenet Evol. 1999;11: 459–476. doi: 10.1006/mpev.1998.0582 10196085 10.1006/mpev.1998.0582

[16] Perelman P, Johnson WE, Roos C, Seuanez HN, Horvath JE, Moreira MAM, et al A molecular phylogeny of living primates. Plos Genet. 2011;7. 10.1371/journal.pgen.1001342 PMC3060065 21436896

[17] Springer MS, Meredith RW, Gatesy J, Emerling CA, Park J, Rabosky DL, et al Macroevolutionary dynamics and historical biogeography of primate diversification inferred from a species supermatrix. Plos One. 2012;7. 10.1371/journal.pone.0049521 PMC3500307 23166696

[18] Schrago CG, Mello B, Soares AER. Combining fossil and molecular data to date the diversification of New World Primates. J Evolution Biol. 2013;26: 2438–2446. 10.1111/jeb.12237 24016177

[19] Byrne H, Rylands AB, Carneiro JC, Alfaro JWL, Bertuol F, da Silva MNF, et al Phylogenetic relationships of the New World titi monkeys (Callicebus): first appraisal of taxonomy based on molecular evidence. Front Zool. 2016;13. 10.1186/s12983-016-0142-4 PMC4774130 26937245

[20] Bouvier M. Biomechanical scaling of mandibular dimensions in New World monkeys. Int J Primatol. 1986;7: 551–567.

[21] Anapol F, Lee S. Morphological adaptation to diet in platyrrhine primates. Am J Phys Anthropol. 1994;94: 239–261. doi: 10.1002/ajpa.1330940208 8085615 10.1002/ajpa.1330940208

[22] Ledogar JA, Winchester JM, St Clair EM, Boyer DM. Diet and dental topography in pitheciine seed predators. Am J Phys Anthropol. 2013;150: 107–121. doi: 10.1002/ajpa.22181 23212472 10.1002/ajpa.22181

[23] van Roosmalen MGM, Mittermeier RA, Milton K. The bearded sakis, genus *Chiropotes* In: Coimbra-Filho AF, Mittermeier RA, editors. Ecology and behavior of Neotropical primates. Rio de Janeiro: Academia Brasiliera de Ciencias; 1981 pp. 419–441.

[24] van Roosmalen MGM, Mittermeier RA, Fleagle JG. Diet of the northern bearded saki (*Chiropotes satanas chiropotes*): a Neotropical seed predator. Am J Primatol. 1988;14: 11–35. 10.1002/ajp.1350140103 32093436

[25] Ayres JM. Comparative feeding ecology of the uakari and bearded saki, *Cacajao* and *Chiropotes*. J Hum Evol. 1989;18: 697–716.

[26] Kinzey WG, Norconk MA. Physical and chemical properties of fruit and seeds eaten by *Pithecia* and *Chiropotes* in Surinam and Venezuela. Int J Primatol. 1993;14: 207–227.

[27] Ayres JM. Uakaris and amazonian flooded forest. Ph.D. Thesis, University of Cambridge. 1986.

[28] Boubli JP. Feeding ecology of black-headed uacaris (*Cacajao melanocephalus melanocephalus*) in Pico da Neblina National Park, Brazil. Int J Primatol. 1999;20: 719–749.

[29] Shaffer CA. Feeding ecology of northern bearded sakis (*Chiropotes sagulatus*) in Guyana. Am J Primatol. 2013;75: 568–580. doi: 10.1002/ajp.22134 23436426 10.1002/ajp.22134

[30] Norconk MA, Conklin-Brittain NL. Variation on frugivory: the diet of Venezuelan white-faced sakis. Int J Primatol. 2004;25: 1–26.

[31] Norconk MA, Wright BW, Conklin-Brittain NL, Vinyard CJ. Mechanical and nutritional properties of food as factors in platyrrhine dietary adaptations In: Garberm PA, Estrada A, Bicca-Marques JC, Heymann EW, Strier KB, editors. South American primates: comparative perspectives in the study of behavior, ecology and conservation. New York: Springer Press; 2009 pp. 279–311.

[32] Norconk MA, Veres M. Physical properties of fruit and seeds ingested by primate seed predators with emphasis on sakis and bearded sakis. Anat Rec. 2011;294: 2092–2111. 10.1002/ar.21506 22042738

[33] Norconk MA, Conklin-Brittain NL. Bearded saki feeding strategies on an island in Lago Guri, Venezuela. Am J Primatol. 2016;78: 507–522. doi: 10.1002/ajp.22396 25809825 10.1002/ajp.22396

[34] Hershkovitz P. The taxonomy of South American sakis, genus *Pithecia* (Cebidae, Platyrrhini): a preliminary report and critical review with the description of a new species and a new subspecies. Am J Primatol. 1987;12: 387–468. 10.1002/ajp.1350120402 31973491

[35] Lucas PW, Teaford MF. Functional morphology of colobine teeth In: Davies AG, Oates JF, editors. Colobine monkeys: their ecology, behaviour and evolution. Cambridge: Cambridge University Press; 1994 pp. 173–203.

[36] Daegling DJ, McGraw WS, Ungar PS, Pampush JD, Vick AE, Bitty EA. Hard-object feeding in sooty mangabeys (*Cercocebus atys*) and interpretation of early hominin feeding ecology. Plos One. 2011;6. 10.1371/journal.pone.0023095 PMC3162570 21887229

[37] Rosenberger AL. The evolution of feeding niches in New World monkeys. Am J Phys Anthropol. 1992;88: 525–562. doi: 10.1002/ajpa.1330880408 1503123 10.1002/ajpa.1330880408

[38] Lucas PW, Luke DA. Chewing it over: basic principles of food breakdown In: Chivers DJ, Wood BA, Bilsborough A, editors. Food acquisition and processing in primates. New York: Plenum Press; 1984 pp. 283–301.

[39] Martin LB, Olejniczak AJ, Maas MC. Enamel thickness and microstructure in pitheciin primates, with comments on dietary adaptations of the middle Miocene hominoid *Kenyapithecus*. J Hum Evol. 2003;45: 351–367. 14624746 10.1016/j.jhevol.2003.08.005

[40] Kinzey WG. The titi monkeys, genus *Callicebus* In: Coimbra-Filho AF, Mittermeier RA, editors. Ecology and behavior of Neotropical primates, Vol 1 Rio de Janeiro: Academia Brasileira de Ciencias; 1981 pp. 241–276.

[41] Palacios E, Rodriguez A, Defler TR. Diet of a group of *Callicebus torquatus lugens* Humboldt, 1812 during the annual resource bottleneck in Amazonian Colombia. Int J Primatol. 1997;18: 503–522.

[42] Fontaine R. The uakaris, genus *Cacajao* In: Coimbra-Filho AF, Mittermeier RA, editors. Ecology and behavior of Neotropical primates. Rio de Janeiro: Academia Brasiliera de Ciencias; 1981 pp. 443–493.

[43] Barnett AA, Brandon-Jones D. The ecology, biogeography and conservation of the uakaris, *Cacajao* (Pitheciinae). Folia Primatol. 1997;68: 223–235.

[44] Barnett AA, de Castilho CV, Shapley RL, Anicacio A. Diet, habitat selection and natural history of *Cacajao melanocephalus ouakary* in Jau National Park, Brazil. Int J Primatol. 2005;26: 949–969.

[45] Fleagle JG. Primate locomotion and diet In: Chivers DJ, Wood BA, Bilsborough A, editors. Food acquisition and processing in primates. New York: Plenum Press; 1984 pp. 105–117.

[46] Mittermeier RA, van Roosmalen MGM. Preliminary observations on habitat utilization and diet in eight Surinam monkeys. Folia Primatol. 1981;36: 1–9. 6802728 10.1159/000156007

[47] Spencer MA. Tooth-root form and function in platyrrhine seed-eaters. Am J Phys Anthropol. 2003;122: 325–335. doi: 10.1002/ajpa.10288 14614754 10.1002/ajpa.10288

[48] Wright BW. Craniodental biomechanics and dietary toughness in the genus *Cebus*. J Hum Evol. 2005;48: 473–492. doi: 10.1016/j.jhevol.2005.01.006 15857651 10.1016/j.jhevol.2005.01.006

[49] Ross CF, Iriarte-Diaz J. What does feeding system morphology tell us about feeding? Evol Anthropol. 2014;23: 105–120. doi: 10.1002/evan.21410 24954218 10.1002/evan.21410

[50] Santana SE, Dumont ER, Davis JL. Mechanics of bite force production and its relationship to diet in bats. Funct Ecol. 2010;24: 776–784.

[51] Peters CR. Nut-like oil seeds: food for monkeys, chimpanzees, humans, and probably ape-men. Am J Phys Anthropol. 1987;73: 333–363. doi: 10.1002/ajpa.1330730306 3113265 10.1002/ajpa.1330730306

[52] Ledogar JA, Benazzi S, Smith AL, Weber GW, Carlson KB, Dechow PC, et al The biomechanics of bony facial "buttresses" in South African australopiths: an experimental study using finite element analysis. Anat Rec. 2017;300: 171–195. 10.1002/ar.23492 28000396

[53] Wroe S, Ferrara TL, McHenry CR, Curnoe D, Chamoli U. The craniomandibular mechanics of being human. P Roy Soc B-Biol Sci. 2010;277: 3579–3586. 10.1098/rspb.2010.0509 PMC2982237 20554545

[54] Ledogar JA, Smith AL, Benazzi S, Weber GW, Spencer MA, Carlson KB, et al Mechanical evidence that *Australopithecus sediba* was limited in its ability to eat hard foods. Nat Commun. 2016;7. 10.1038/ncomms10596 PMC4748115 26853550

[55] Dumont ER, Davis JL, Grosse IR, Burrows AM. Finite element analysis of performance in the skulls of marmosets and tamarins. J Anat. 2011;218: 151–162. doi: 10.1111/j.1469-7580.2010.01247.x 20572898 10.1111/j.1469-7580.2010.01247.x PMC3039787

[56] Ross CF, Berthaume MA, Dechow PC, Iriarte-Diaz J, Porro LB, Richmond BG, et al *In vivo* bone strain and finite-element modeling of the craniofacial haft in catarrhine primates. J Anat. 2011;218: 112–141. doi: 10.1111/j.1469-7580.2010.01322.x 21105871 10.1111/j.1469-7580.2010.01322.x PMC3039785

[57] Smith AL, Benazzi S, Ledogar JA, Tamvada K, Smith LCP, Weber GW, et al Biomechanical implications of intraspecific shape variation in chimpanzee crania: moving toward an integration of geometric morphometrics and finite element analysis. Anat Rec. 2015;298: 122–144. 10.1002/ar.23074 PMC4274755 25529239

[58] Panagiotopoulou O, Iriarte-Diaz J, Wilshin S, Dechow PC, Taylor AB, Mehari Abraha H, et al *In vivo* bone strain and finite element modeling of a rhesus macaque mandible during mastication. Zoology (Jena). 2017;124: 13–29. 29037463 10.1016/j.zool.2017.08.010 PMC5792078

[59] Demes B, Creel N. Bite force, diet, and cranial morphology of fossil hominids. J Hum Evol. 1988;17: 657–670.

[60] White T, Black M, Folkens P. Human Osteology (3rd edition). San Diego: Academic Press; 2011.

[61] Baab KL. Cranial shape variation in *Homo erectus*. Ph.D. Thesis, City University of New York. 2007.

[62] Gunz P, Mitteroecker P. Semilandmarks: a method for quantifying curves and surfaces. Hystrix. 2013;24: 103–109.

[63] Bookstein FL. Morphometric tools for landmark data: geometry and biology New York: Cambridge University Press; 1991.

[64] Slice DE. Modern morphometrics In: Slice DE, editor. Modern morphometrics in physical anthropology. Dordrecht: Kluwer Academic Publishers; 2005 pp. 1–45.

[65] R Core Team. R: A language and environment for statistical computing. Vienna, Austria. 2017.

[66] Adams DC, Collyer ML, Kaliontzopoulou A, Sherratt E. geomorph: software for geometric morphometric analyses. R package version 3.0.4. 2017.

[67] Ledogar JA, Dechow PC, Wang Q, Gharpure PH, Gordon AD, Baab KL, et al Human feeding biomechanics: performance, variation, and functional constraints. Peerj. 2016;4: e2242 doi: 10.7717/peerj.2242 27547550 10.7717/peerj.2242 PMC4975005

[68] Marcé-Nogué J, Püschel TA, Kaiser TM. A biomechanical approach to understand the ecomorphological relationship between primate mandibles and diet. Scientific Reports. 2017;7: 8364 doi: 10.1038/s41598-017-08161-0 28827696 10.1038/s41598-017-08161-0 PMC5567063

[69] Wood SA, Strait DS, Dumont ER, Ross CF, Grosse IR. The effects of modeling simplifications on craniofacial finite element models: The alveoli (tooth sockets) and periodontal ligaments. J Biomech. 2011;44: 1831–1838. doi: 10.1016/j.jbiomech.2011.03.022 21592483 10.1016/j.jbiomech.2011.03.022

[70] Magne P. Efficient 3D finite element analysis of dental restorative procedures using micro-CT data. Dent Mater. 2007;23: 539–548. doi: 10.1016/j.dental.2006.03.013 16730058 10.1016/j.dental.2006.03.013

[71] Ko CC, Chu CS, Chung KH, Lee MC. Effects of posts on dentin stress-distribution in pulpless teeth. J Prosthet Dent. 1992;68: 421–427. 1432755 10.1016/0022-3913(92)90404-x

[72] Rubin C, Krishnamurthy N, Capilouto E, Yi H. Stress analysis of the human tooth using a three-dimensional finite element model. J Dent Res. 1983;62: 82–86. doi: 10.1177/00220345830620021701 6571871 10.1177/00220345830620021701

[73] Holmes DC, DiazArnold AM, Leary JM. Influence of post dimension on stress distribution in dentin. J Prosthet Dent. 1996;75: 140–147. 8667271 10.1016/s0022-3913(96)90090-6

[74] Strait DS, Grosse IR, Dechow PC, Smith AL, Wang Q, Weber GW, et al The structural rigidity of the cranium of *Australopithecus africanus*: implications for diet, dietary adaptations, and the allometry of feeding biomechanics. Anat Rec. 2010;293: 583–593. 10.1002/ar.21122 20235314

[75] Anapol F, Shahnoor N, Ross CF. Scaling of reduced physiologic cross-sectional area in primate muscles of mastication In: Vinyard C, Ravosa MJ, Wall CE, editors. Primate craniotacial function and biology. New York: Springer; 2008 pp. 201–216.

[76] Murphy RA. Skeletal muscle In: Berne RM, Levy MN, editors. Physiology. St. Louis: Mosby; 1998.

[77] Dumont ER, Grosse IR, Slater GJ. Requirements for comparing the performance of finite element models of biological structures. J Theor Biol. 2009;256: 96–103. doi: 10.1016/j.jtbi.2008.08.017 18834892 10.1016/j.jtbi.2008.08.017

[78] Cachel SM. A functional analysis of the primate masticatory system and the origin of the anthropoid post-orbital septum. Am J Phys Anthropol. 1979;50: 1–18. 10.1002/ajpa.1330500102 104630

[79] Taylor AB, Yuan T, Ross CF, Vinyard CJ. Jaw-muscle force and excursion scale with negative allometry in platyrrhine primates. Am J Phys Anthropol. 2015;158: 242–256. 10.1002/ajpa.22782 26175006

[80] Starck D. Die Kaumuskulatur der Platyrrhinen. Gegenbaurs Morphologisches Jahrbuch. 1933;72: 212–285.

[81] Grosse IR, Dumont ER, Coletta C, Tolleson A. Techniques for modeling muscle-induced forces in finite element models of skeletal structures. Anat Rec. 2007;290: 1069–1088. 10.1002/ar.20568 17721980

[82] Perry JMG, Bastian ML, St Clair E, Hartstone-Rose A. Maximum ingested food size in captive anthropoids. Am J Phys Anthropol. 2015;158: 92–104. doi: 10.1002/ajpa.22779 26119490 10.1002/ajpa.22779

[83] Perry JM, Hartstone-Rose A, Logan RL. The jaw adductor resultant and estimated bite force in primates. Anat Res Int. 2011;2011: 929848 doi: 10.1155/2011/929848 22611496 10.1155/2011/929848 PMC3349241

[84] Hylander WL, Picq PG, Johnson KR. Masticatory stress hypotheses and the supraorbital region of primates. Am J Phys Anthropol. 1991;86: 1–36. doi: 10.1002/ajpa.1330860102 1951658 10.1002/ajpa.1330860102

[85] Hylander WL, Johnson KR. *In vivo* bone strain patterns in the zygomatic arch of macaques and the significance of these patterns for functional interpretations of craniofacial form. Am J Phys Anthropol. 1997;102: 203–232. doi: 10.1002/(SICI)1096-8644(199702)102:2<203::AID-AJPA5>3.0.CO;2-Z 9066901 10.1002/(SICI)1096-8644(199702)102:2<203::AID-AJPA5>3.0.CO;2-Z

[86] Greaves WS. Jaw lever system in ungulates: A new model. J Zool. 1978;184: 271–285.

[87] Spencer MA. Constraints on masticatory system evolution in anthropoid primates. Am J Phys Anthropol. 1999;108: 483–506. doi: 10.1002/(SICI)1096-8644(199904)108:4<483::AID-AJPA7>3.0.CO;2-L 10229390 10.1002/(SICI)1096-8644(199904)108:4<483::AID-AJPA7>3.0.CO;2-L

[88] Antón SC. Cranial adaptation to a high attrition diet in Japanese macaques. Int J Primatol. 1996;17: 401–427.

[89] Koyabu DB, Endo H. Craniodental mechanics and diet in Asian colobines: morphological evidence of mature seed predation and sclerocarpy. Am J Phys Anthropol. 2010;142: 137–148. doi: 10.1002/ajpa.21213 20091848 10.1002/ajpa.21213

[90] Endo B. Experimental studies on the mechanical significance of the form of the human facial skeleton. J Faculty of Sci. 1966;3: 1–106.

[91] Prado FB, Freire AR, Rossi AC, Ledogar JA, Smith AL, Dechow PC, et al Review of *in vivo* bone strain studies and finite element models of the zygomatic complex in humans and nonhuman primates: implications for clinical research and practice. Anat Rec. 2016;299: 1753–1778. 10.1002/ar.23486 27870351

[92] Eisenberg NA, Brodie AG. Antagonism of temporal fascia to masseteric contraction. Anat Rec. 1965;152: 185–192. 4954088 10.1002/ar.1091520209

[93] Curtis N, Witzel U, Fitton L, O'Higgins P, Fagan M. The mechanical significance of the temporal fasciae in *Macaca fascicularis*: an investigation using finite element analysis. Anat Rec. 2011;294: 1178–1190. 10.1002/ar.21415 21618443

[94] Ross CF, Patel BA, Slice DE, Strait DS, Dechow PC, Richmond BG, et al Modeling masticatory muscle force in finite element analysis: sensitivity analysis using principal coordinates analysis. Anat Rec Part A. 2005;283a: 288–299. 10.1002/ar.a.20170 15747351

[95] Strait DS, Wang Q, Dechow PC, Ross CF, Richmond BG, Spencer MA, et al Modeling elastic properties in finite element analysis: how much precision is needed to produce an accurate model? Anat Rec Part A. 2005;283a: 275–287. 10.1002/ar.a.20172 15747346

[96] Ross CF. *In vivo* function of the craniofacial haft: The interorbital "pillar". Am J Phys Anthropol. 2001;116: 108–139. doi: 10.1002/ajpa.1106 11590585 10.1002/ajpa.1106

[97] Janson CH. Intraspecific food competition and primate social structure: a synthesis. Behaviour. 1988;105: 1–17.

[98] Kamilar JM, Ledogar JA. Species co-occurrence patterns and dietary resource competition in primates. Am J Phys Anthropol. 2011;144: 131–139. doi: 10.1002/ajpa.21380 20740497 10.1002/ajpa.21380

[99] Baab KL, Perry JMG, Rohlf FJ, Jungers WL. Phylogenetic, ecological, and allometric correlates of cranial shape in Malagasy lemuriforms. Evolution. 2014;68: 1450–1468. doi: 10.1111/evo.12361 24451053 10.1111/evo.12361

[100] Ross CF, Iriarte-Diaz J, Nunn CL. Innovative approaches to the relationship between diet and mandibular morphology in primates. Int J Primatol. 2012;33: 632–660.

[101] Ledogar JA. Functional analysis of craniomandibular morphology in durophagous, folivorous, and sclerocarpic harvesting anthropoids. M.A. Thesis, Stony Brook University. 2009.

[102] Spencer MA, Demes B. Biomechanical analysis of masticatory system configuration in Neandertals and Inuits. Am J Phys Anthropol. 1993;91: 1–20. doi: 10.1002/ajpa.1330910102 8512051 10.1002/ajpa.1330910102

[103] Carter K, Worthington S. Morphologic and demographic predictors of third molar agenesis: a systematic review and meta-analysis. J Dent Res. 2015;94: 886–894. doi: 10.1177/0022034515581644 25883107 10.1177/0022034515581644

[104] Lieberman DE. The evolution of the human head Cambridge: Belknap Press; 2011.

[105] Heymann EW, Aquino R. Peruvian red uakaris (*Cacajao calvus ucayalii*) are not flooded-forest specialists. Int J Primatol. 2010;31: 751–758. doi: 10.1007/s10764-010-9425-3 20949117 10.1007/s10764-010-9425-3 PMC2945472

[106] Bowler M, Bodmer R. Social behavior in fission-fusion groups of red uakari monkeys (*Cacajao calvus ucayalii*). Am J Primatol. 2009;71: 976–987. doi: 10.1002/ajp.20740 19722260 10.1002/ajp.20740

[107] Lambert JE, Chapman CA, Wrangham RW, Conklin-Brittain NL. Hardness of cercopithecine foods: implications for the critical function of enamel thickness in exploiting fallback foods. Am J Phys Anthropol. 2004;125: 363–368. doi: 10.1002/ajpa.10403 15386250 10.1002/ajpa.10403

[108] Dominy NJ, Vogel ER, Yeakel JD, Constantino P, Lucas PW. Mechanical properties of plant underground storage organs and implications for dietary models of early hominins. Evol Biol. 2008;35: 159–175.

[109] Vogel ER, van Woerden JT, Lucas PW, Atmoko SSU, van Schaik CP, Dominy NJ. Functional ecology and evolution of hominoid molar enamel thickness: *Pan troglodytes schweinfurthii* and *Pongo pygmaeus wurmbii*. J Hum Evol. 2008;55: 60–74. doi: 10.1016/j.jhevol.2007.12.005 18243275 10.1016/j.jhevol.2007.12.005

[110] Yamashita N, Vinyard CJ, Tan CL. Food mechanical properties in three sympatric species of *Hapalemur* in Ranomafana National Park, Madagascar. Am J Phys Anthropol. 2009;139: 368–381. doi: 10.1002/ajpa.20992 19115398 10.1002/ajpa.20992

[111] Lucas PW, Copes L, Constantino PJ, Vogel ER, Chalk J, Talebi M, et al Measuring the toughness of primate foods and its ecological value. Int J Primatol. 2012;33: 598–610.

[112] Wright BW, Willis MS. Relationships between the diet and dentition of Asian leaf monkeys. Am J Phys Anthropol. 2012;148: 262–275. doi: 10.1002/ajpa.22081 22610901 10.1002/ajpa.22081

[113] McGraw WS, Vick AE, Daegling DJ. Dietary variation and food hardness in sooty mangabeys (*Cercocebus atys*): implications for fallback foods and dental adaptation. Am J Phys Anthropol. 2014;154: 413–423. doi: 10.1002/ajpa.22525 24810136 10.1002/ajpa.22525

[114] Hartstone-Rose A, Parkinson JA, Criste T, Perry JMG. Comparing apples and oranges: the influence of food mechanical properties on ingestive bite sizes in lemurs. Am J Phys Anthropol. 2015;157: 513–518. doi: 10.1002/ajpa.22726 25727399 10.1002/ajpa.22726

[115] Chalk J, Wright BW, Lucas PW, Schuhmacher KD, Vogel ER, Fragaszy D, et al Age-related variation in the mechanical properties of foods processed by *Sapajus libidinosus*. Am J Phys Anthropol. 2016;159: 199–209. doi: 10.1002/ajpa.22865 26381730 10.1002/ajpa.22865

[116] Dunham NT, Lambert AL. The role of leaf toughness on foraging efficiency in Angola black and white colobus monkeys (*Colobus angolensis palliatus*). Am J Phys Anthropol. 2016;161: 343–354. doi: 10.1002/ajpa.23036 27346431 10.1002/ajpa.23036

[117] Glowacka H, McFarlin SC, Vogel ER, Stoinski TS, Ndagijimana F, Tuyisingize D, et al Toughness of the Virunga mountain gorilla (*Gorilla beringei beringei*) diet across an altitudinal gradient. Am J Primatol. 2017;79. 10.1002/ajp.22661 28388822

[118] Lucas PW. Dental functional morphology Cambridge Cambridge University Press; 2004.

